# Automated solvent-assisted flavor evaporation 2.0 (aSAFE 2.0)

**DOI:** 10.1016/j.mex.2026.104061

**Published:** 2026-07-24

**Authors:** Klaas Reglitz, Martin Steinhaus

**Affiliations:** Leibniz Institute for Food Systems Biology at the Technical University of Munich (Leibniz-LSB@TUM), Freising, Germany

**Keywords:** aSAFE 2.0, Volatile isolation, High vacuum, Artifact avoidance, Gas chromatography-olfactometry (GC–O)

## Abstract

Before using gas chromatography to analyze volatiles in food, beverages, or other materials, it is necessary to carefully separate the volatile fraction from the nonvolatile compounds. Mild approaches that avoid elevated temperatures such as the solvent-assisted flavor evaporation are key to prevent compositional changes associated with compound degradation and artifact formation. This paper introduces a novel system for the automated execution of a solvent-assisted flavor evaporation that combines a newly designed glassware with a novel light-weight valve, a novel modernized controller, and an endpoint detection sensor. In summary, the major advantages of the novel aSAFE 2.0 system for the isolation of volatiles in the lab are:

• Representativeness of the volatile isolates: no thermal compound degradation or artifact formation, high yields over a wide range of boiling points
• Purity of the volatile isolates: reduced risk of unintended transfers of nonvolatiles
• Convenience of handling: easy programming, autonomous execution, automatic shutdown

## Graphical abstract

**Figure:**
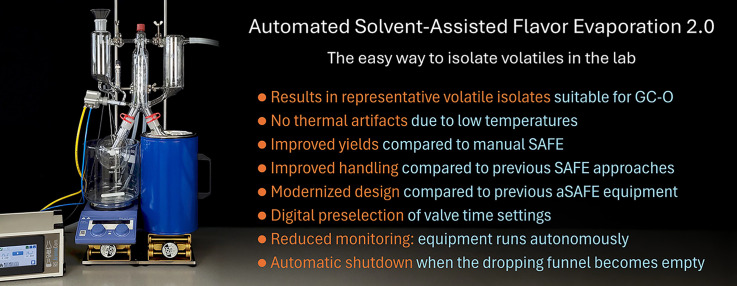

## Specifications table

**Table:** 

Subject area	Food Science
More specific subject area	Food Aroma Research
Name of your method	Automated solvent-assisted flavor evaporation 2.0 (aSAFE 2.0)
Name and reference of original method	1. P. Schlumpberger, C.A. Stübner, M. Steinhaus, Development and evaluation of an automated solvent‑assisted flavour evaporation (aSAFE), Eur. Food Res. Technol. 248 (2022) 2591–2602. https://doi.org/10.1007/s00217-022-04072-1 2. W. Engel, W. Bahr, P. Schieberle, Solvent assisted flavour evaporation – a new and versatile technique for the careful and direct isolation of aroma compounds from complex food matrices, Eur. Food Res. Technol. 209 (1999) 237–241. https://doi.org/10.1007/s002170050486
Resource availability	aSAFE 2.0 Kit: RS AromaCom, Am Lichtfeld 9, 85386 Eching, Germany; RSAromaCom@gmail.com; the kit includes the aSAFE 2.0 glass device, the pneumatic valve, the endpoint detection sensor, and the controller

## Background

Separation of the volatile fraction from the nonvolatile fraction is a crucial step before analyzing food volatiles by gas chromatography. However, harsh approaches, foremost those involving heat (e.g., hydrodistillation at ambient pressure, direct injection of solvent extracts into hot GC injectors, microextraction approaches in combination with thermal desorption, etc.), have been shown to lead to artifact formation [[1], [2], [3], [4], [5], [6]]. This is a particular problem in aroma research: due to an enormous range of odor threshold concentrations (OTCs) [7], even a minor formation of highly odor-active artifacts can be sufficient to substantially falsify results, notably in activity-guided odorant screening approaches using gas chromatography-olfactometry (GC–O) [8].

In 1970, Weurman et al. introduced an artifact-avoiding high-vacuum transfer (HVT) approach for the direct isolation of food volatiles at ambient temperature [9]. Grosch, Schieberle, and coworkers adopted the approach to low-boiling solvent extracts [10], improved volatile recoveries by using moderately thermostated double-walled glassware [11], and converted the static into a dynamic approach, in which the extract is introduced into the high vacuum in discrete portions by using a dropping funnel, which further improved recoveries [12], thereby ensuring effective retention of nonvolatiles with a splash protection adapter [13]. In 1999, Engel et al. combined the crucial glassware components into a single device with further improvement of thermostatization, nonvolatile retention, volatile yield, and handling, and named the approach solvent-assisted flavor evaporation (SAFE) [14]. For the next two decades, SAFE became a worldwide standard for the artifact-avoiding isolation of volatiles from complex food matrices, particularly in aroma research.

In 2022, our group developed an automated SAFE approach (aSAFE) by replacing the manual valve of the device’s dropping funnel with a normally closed pneumatic valve connected to a programmable electronic controller, which resulted in further improvements [15]: Foremost, automatization allowed reducing the volume of individual extract portions, which, together with longer intervals between the portions, resulted in higher yields, particularly from high-fat extracts and for volatiles with elevated boiling points. Moreover, aSAFE excluded unintended transfers of nonvolatiles associated with large extract portions, which often spoiled workups in the manual approach, and reduced lab manpower requirements. We additionally demonstrated that even full automation was possible when combining the aSAFE approach with an automated liquid nitrogen refill system and an endpoint detection and shut-off system.

**Fig. 1:**
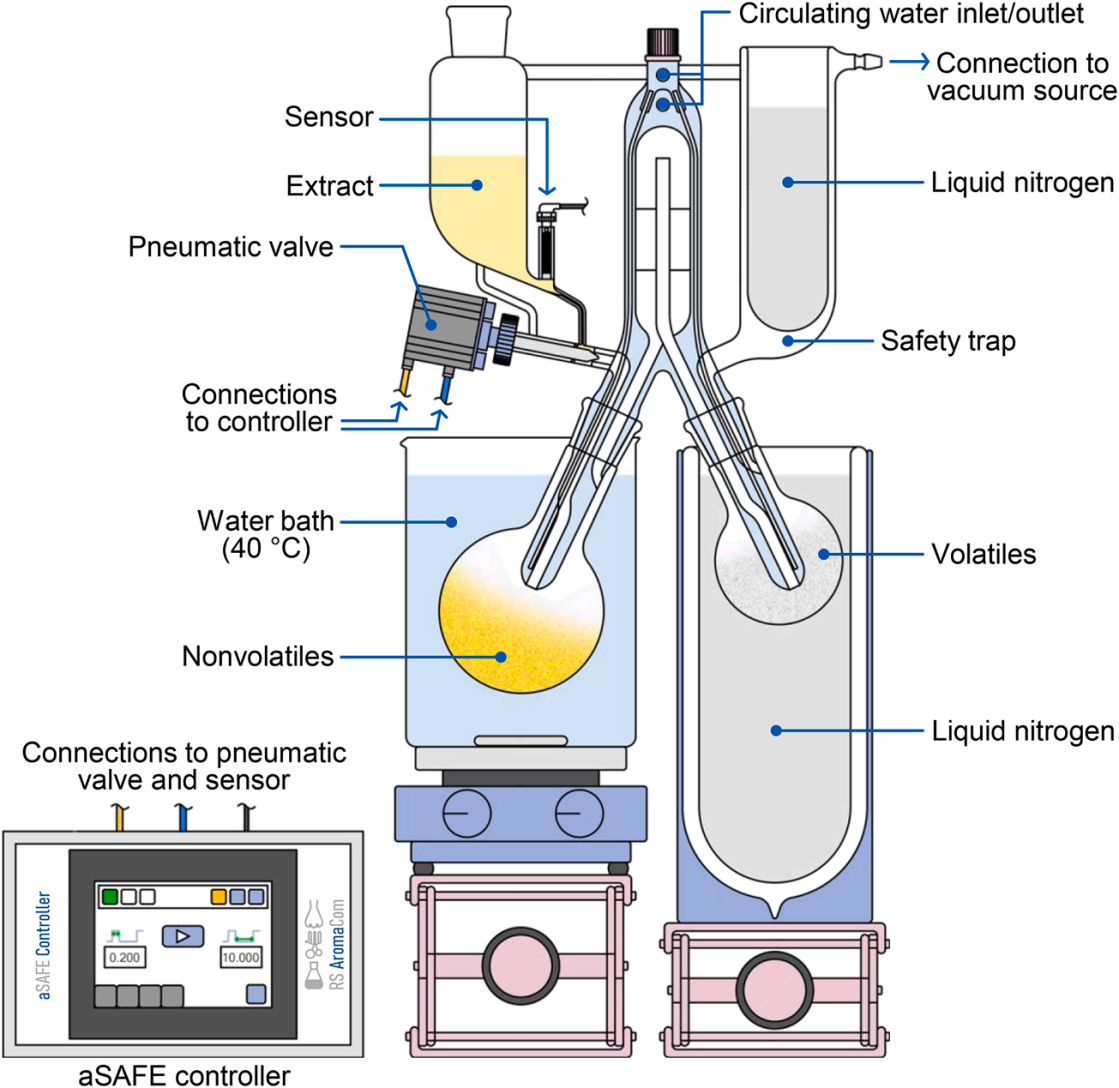
The aSAFE 2.0 system including the modified glassware with the endpoint detection sensor, the new valve design, and the novel, modernized valve control unit.

While aSAFE became the standard in our labs and has been successfully applied in numerous projects since [e.g., [16], [17], [18], [19], [20], [21]], further spreading of the approach, despite great interest in the food aroma research community, was unfortunately hindered, because the supplier of the valve controller soon removed it from its product portfolio. This prompted us to look for alternative and more reliable suppliers. In this paper, we present the aSAFE 2.0 approach, which comes not only with a novel, modernized controller, but also with a lighter version of the pneumatic valve and a slightly modified glassware allowing for the integration of a capacitive endpoint detection sensor directly connected to the controller, whereas we decided against automated nitrogen refilling to reduce complexity (Fig. 1).

## Method details

### Required resources and materials

**Fig. 2:**
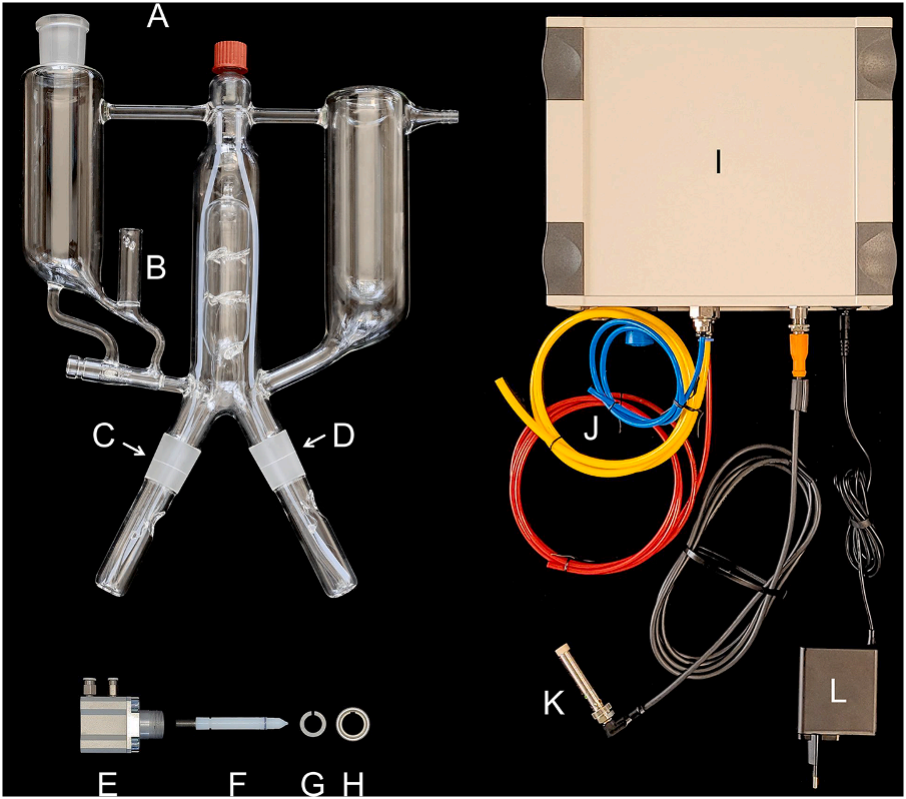
The aSAFE 2.0 kit including the aSAFE 2.0 glass device (A–D), the pneumatic valve (E–H), and the controller (I–L).
• A high-vacuum source with a pumping speed of ∼60–80 L/s and an ultimate pressure of ∼10−5 Pa such as a T-Station 85 (Edwards, Burgess Hill, UK), a TURBOLAB 80 (Leybold, Cologne, Germany), or a HiCube 80 Neo (Pfeiffer Vacuum, Aßlar, Germany); make sure that the high-vacuum source is equipped with a pressure-gauge and a lock valve (an additional vent valve is optional); the connection to the aSAFE 2.0 glass device requires a hose connector that fits a silicone vacuum tubing, 16 mm × 8 mm (o.d. × i.d.)
• A source of compressed air (∼300 kPa; however, any pressure between 200 and 600 kPa is acceptable)
• Liquid nitrogen
• A water-filled circulation thermostat suitable for a temperature of 40 °C
• A hot-plate magnetic stirrer equipped with an external temperature sensor
• A cylindrical Dewar flask, approx. 30 cm × 14 cm (height × i.d.)
• Two adjustable lab jacks, platform size approx. 20 × 20 cm
• Silicone tubing, 9 mm × 6 mm (o.d. × i.d.)
• Silicone vacuum tubing, 16 mm × 8 mm (o.d. × i.d.)
• A beaker, borosilicate glass, approx. 16 cm × 16 cm (height × i.d.)
• A round-bottom flask, borosilicate glass, 250 mL, ST/NS 29/32
• A round-bottom flask, borosilicate glass, 500 mL, ST/NS 29/32
• Two joint clips for ST/NS 29 joints
• A magnetic stir bar, approx. 60 mm × 10 mm (length × diameter)
• A magnetic stir bar, approx. 25 mm × 5 mm (length × diameter)

• Personnel familiar with working in a chemical laboratory, particularly with regard to the handling of organic solvents, liquid nitrogen, and high-vacuum systems
• Personal protective equipment (PPE), including safety glasses, lab coat, and cryo gloves for liquid nitrogen handling
• Bench space of approx. 120 cm wide and 60 cm deep in a fume hood equipped with a lattice system and appropriate ventilation
• The aSAFE 2.0 Kit as listed in the specifications table (Fig. 2)

### Setting up the aSAFE 2.0 equipment

Before you start, check your PPE according to the requirements applicable in your lab. At least, wear safety glasses and a lab coat, and have your cryo gloves ready for liquid nitrogen handling.

**Fig. 3:**
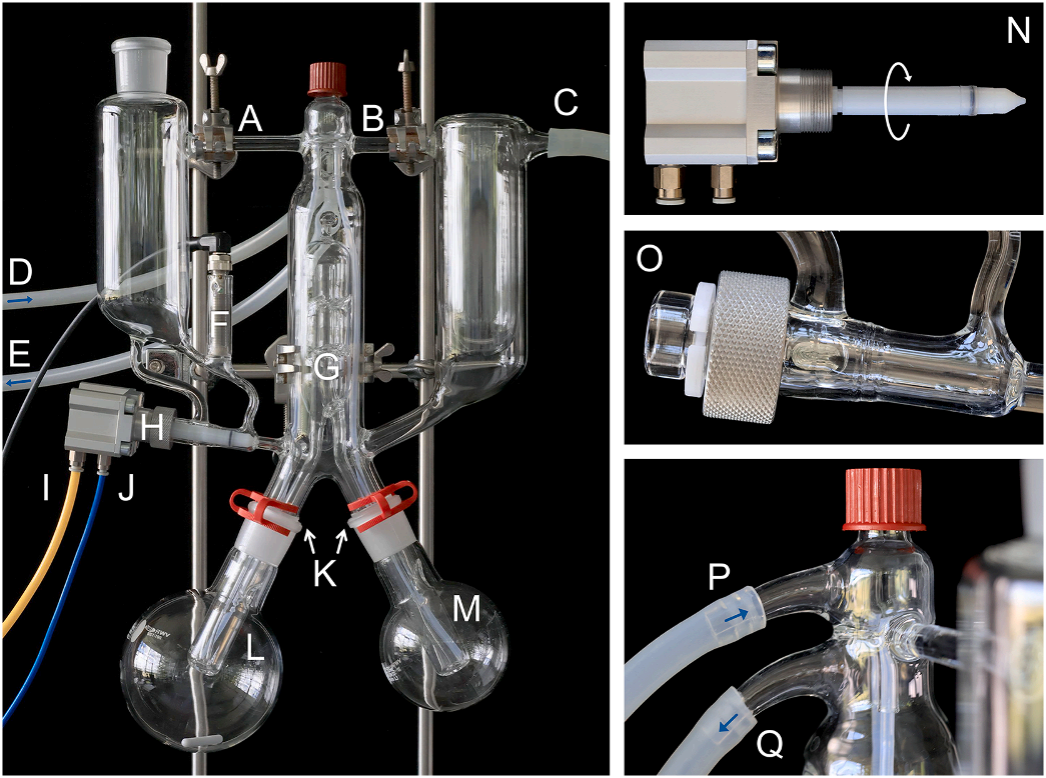
Setting up the aSAFE 2.0 equipment.
• Screw the PTFE plunger (Fig. 2F) into the internal thread of the pneumatic valve (Fig. 2E) as shown in Fig. 3N
• Slide the union nut (Fig. 2H) onto the plunger casing of the aSAFE 2.0 glass device and secure it by inserting the split washer (Fig. 2G) into the groove (Fig. 3O)
• Attach the pneumatic valve to the aSAFE 2.0 glass device by inserting the plunger into its casing and tightening the union nut (Fig. 3H)
• Attach the aSAFE 2.0 glass device to the lattice system using appropriate sockets and clamps, pneumatic valve and dropping funnel on the left, safety cold trap on the right (Fig. 3); refer to Fig. 3A, B, and G for the best attachment points
• Connect the valve to the aSAFE controller; connect the larger valve inlet to the yellow pneumatic tubing (Fig. 3I) and the smaller valve inlet to the blue pneumatic tubing (Fig. 3J)
• Insert the endpoint detection sensor into its casing on the aSAFE 2.0 glass device (Figs. 2B and 3F)
• Connect the aSAFE 2.0 glass device to the circulation thermostat using the thin silicone tubing (Fig. 3D, E); the upper hose barb of the aSAFE 2.0 glass device is the inlet, the lower hose barb is the outlet; swapping the two can cause the circulating water to freeze and destroy the aSAFE 2.0 glass device
• Connect the aSAFE 2.0 glass device to the high-vacuum source using the thick silicone vacuum tubing (Fig. 3C)
• Add the large stir bar to the beaker (Fig. 4J), put it on the hot-plate magnetic stirrer (Fig. 4K) placed on a lab jack (Fig. 4L) below the evaporation flask (Fig. 2 L and 4H), and add water until just below the ground joint (Fig. 4G)

• Check the aSAFE 2.0 glass device (Fig. 2A) for cracks; if you notice a crack—no matter how small it may be, or even if you are unsure—do not use the device; instead, contact the supplier for repair or replacement; for safety reasons, an aSAFE 2.0 glass device with cracks must not be exposed to a vacuum under any circumstances
• Ensure that the two PTFE sealing rings (Fig. 2C, D) are fitted to the aSAFE 2.0 glass device
• Check the 250 and 500 mL round-bottom flasks for cracks; if you notice a crack—no matter how small it may be, or even if you are unsure—discard the flask and replace it with a new one that is free of cracks; for safety reasons, flasks with cracks must not be exposed to a vacuum under any circumstances
• Add the small stir bar to the 500 mL round-bottom flask; connect this flask to the side of the aSAFE 2.0 glass device with the dropping funnel (Fig. 3L); connect the 250 mL round-bottom flask to the other side of the aSAFE 2.0 glass device (Fig. 3M); secure the flasks with the joint clips (Fig. 3K)

**Fig. 4:**
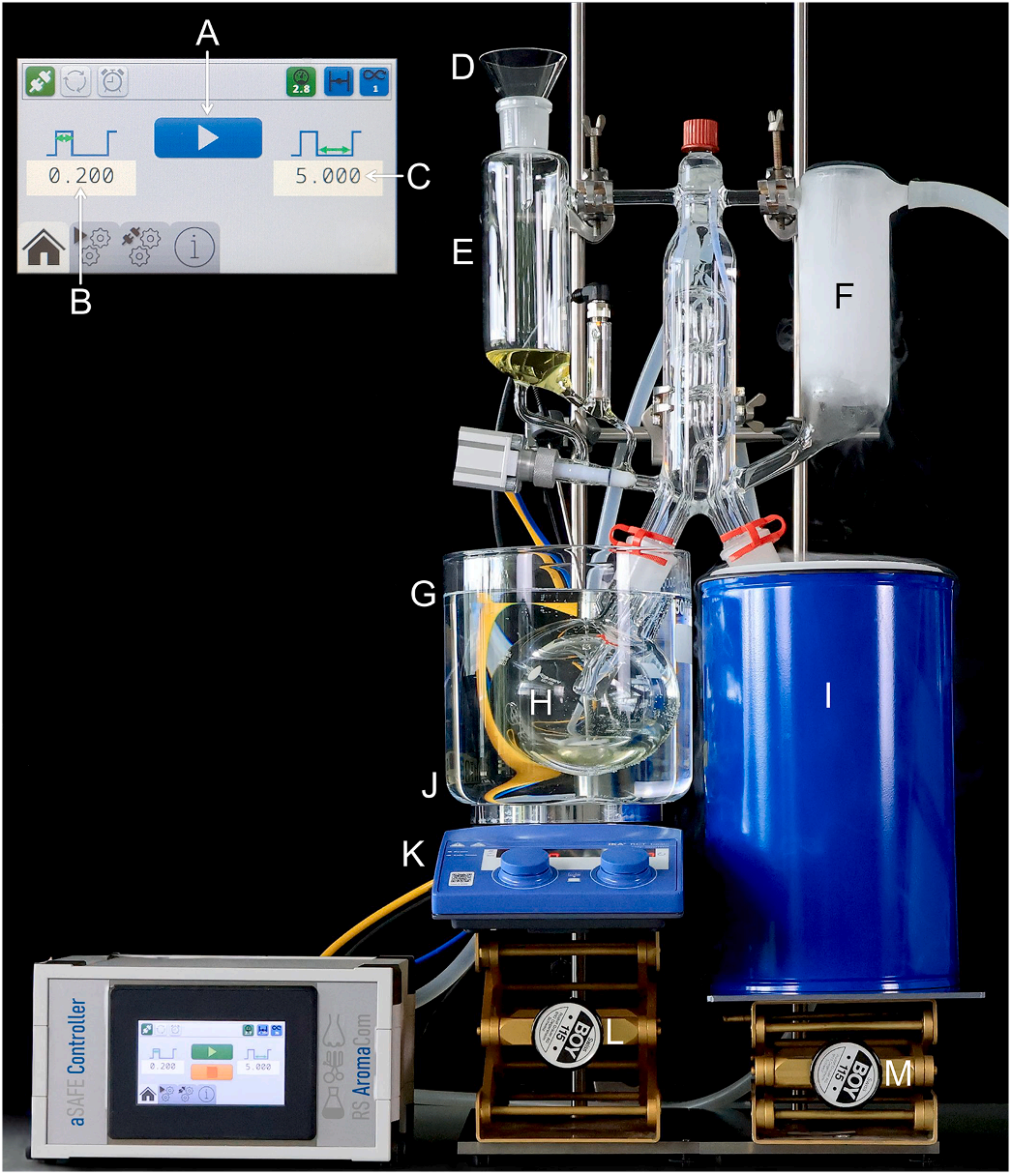
Using the aSAFE 2.0 equipment.
• Connect the external temperature sensor to the hot-plate magnetic stirrer and insert it into the water bath
• Set the hot-plate magnetic stirrer to 40 °C and switch it on
• Place the empty Dewar flask (Fig. 4I) on a lab jack (Fig. 4M) below the recondensation flask (Fig. 3M)
• Close the fume hood window
• Evacuate the aSAFE 2.0 equipment; consider the operating instructions of the high-vacuum source instrument
• When the pressure gauge reads a value below 10^–2^ Pa, set the circulation thermostat to 40 °C and switch it on
• When the circulating water has reached 40 °C, check your PPE for liquid nitrogen handling (safety glasses, cryo gloves) and add liquid nitrogen to the Dewar flask (Fig. 4I) until just below the ground joint as well as to the safety cold trap (Fig. 4F) of the aSAFE 2.0 glass device

The aSAFE 2.0 equipment is now ready for processing a sample. The sample is typically a dried (e.g., above anhydrous sodium sulfate) and filtered solvent extract obtained from a food, beverage, or any other material. Most frequently applied organic solvents include diethyl ether and dichloromethane [8].

### Using the aSAFE 2.0 equipment to isolate the volatile fraction from foods or beverages

• Double-check the vacuum (≤ 10^–2^ Pa) and the temperatures of the circulating water and the water bath (target temperature 40 °C)
• Pour the sample into the dropping funnel (Fig. 4E) of the aSAFE 2.0 glass device; consider using a glass funnel (Fig. 4D)
• Make sure the area between the bottom of the dropping funnel and the valve is free of air bubbles; otherwise, the endpoint detection sensor may prevent the system from starting; air bubbles can be removed by gently tapping or slightly tilting the device
• Switch on the aSAFE controller and enter the setpoints for the open time (Fig. 4B) and the closed time (Fig. 4C) of the pneumatic valve; if you are unsure about the appropriate settings, try a combination of 0.2 s and 20 s first; however, ensure complete evaporation of the solvent of an extract portion before the next portion enters (visually) as well as complete recondensation/solidification (indicated by smoothening of the surface of the liquid nitrogen in the Dewar flask)
• Start the aSAFE 2.0 procedure by tapping the play icon **▹** on the screen of the aSAFE controller (Fig. 4A)
• Watch the Dewar flask (Fig. 4I) and the safety cold trap (Fig. 4F) of the aSAFE 2.0 glass device, and add liquid nitrogen from time to time to maintain appropriate cooling; however, make sure that the liquid nitrogen level in the Dewar flask remains below the ground joint

When the dropping funnel is empty and air enters the area monitored by the endpoint detection sensor between the dropping funnel and the valve, the aSAFE controller will stop the aSAFE 2.0 procedure. The system remains in a stable state as long as the cold traps provide sufficient cooling—that is, as long as liquid nitrogen is present—which offers the operator some flexibility regarding when to collect the distillate.

**Fig. 5:**
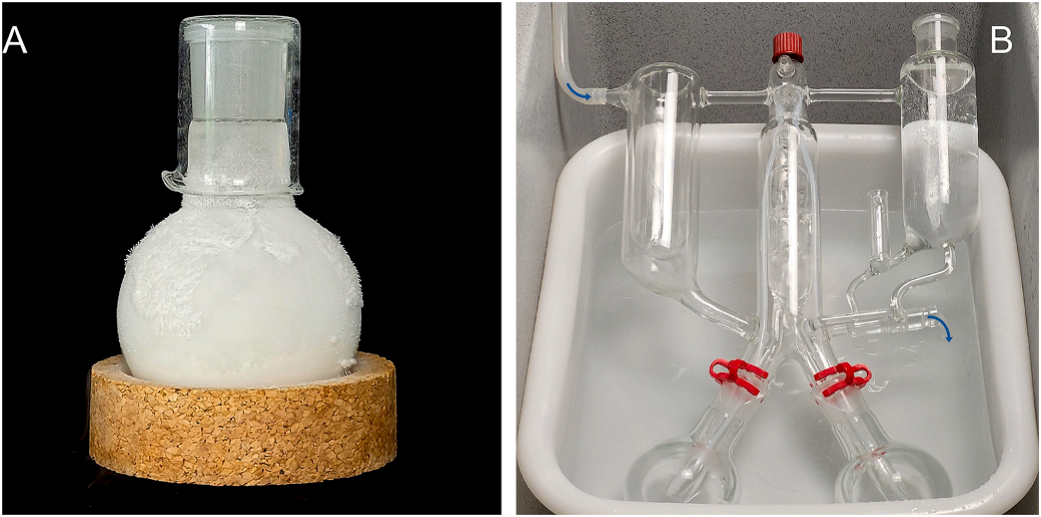
A: The result of the aSAFE 2.0 process: an isolated volatile fraction during thawing; B: Cleaning the aSAFE 2.0 glass device.

• Finally, remove the Dewar flask
• Carefully dispose of the remaining liquid nitrogen from the Dewar flask; for safety reasons, avoid contact of the liquid nitrogen residues with combustable materials and do not reuse them, as oxygen may have condensed into it
• Disconnect the high vacuum source
• Carefully vent the system
• Remove the recondensation flask and let the volatile isolate thaw in a fume hood (Fig. 5A) before further workup and analysis [8]; under no circumstances should you seal the flask with a stopper while thawing, as pressure buildup could cause the flask to burst; instead, consider placing a small beaker upside down over the flask to prevent moisture in the ambient air from entering without the risk of the flask bursting

### Shutdown, cleaning, and storage of the aSAFE 2.0 equipment

• Switch off the aSAFE 2.0 controller
• Disconnect the pneumatic tubings from the pneumatic valve, then disconnect the valve from the aSAFE 2.0 glass device
• Unscrew the plunger from the pneumatic valve and clean it separately
• Disconnect the endpoint detection sensor from the aSAFE 2.0 glass device
• Switch off the hot-plate magnetic stirrer; remove the hot-plate magnetic stirrer, the connected external temperature sensor, and the water bath
• Switch off the circulation thermostat; disconnect all silicone tubing from the aSAFE 2.0 glass device
• Disconnect the aSAFE 2.0 glass device from the lattice system and carefully dispose of the remaining liquid nitrogen from the safety cold trap; for safety reasons, avoid contact of the liquid nitrogen residues with combustable materials and do not reuse them, as oxygen may have condensed into it
• Remove the evaporation flask and clean it separately
• Clean the aSAFE 2.0 glass device; the interior can be cleaned effectively by rinsing it with hot water and a small amount of unscented detergent from the vacuum connection side while two 250 mL round-bottom flasks are attached to the legs of the device (Fig. 5B)
• Finally, rinse the aSAFE 2.0 glass device with pure water, and dry and store it in a drying cabinet at 105–120 °C; before next use, carefully remove the hot device from the drying cabinet and let it come to room temperature before attaching it to the lattice system

### Variants

• **Mirrored orientation**

The aSAFE 2.0 glass device may also be installed in a mirrored orientation; however, this will result in the connections to the circulating thermostat pointing forward.

• **aSAFE 2.0 without endpoint detection**

The aSAFE controller does not necessarily require the endpoint detection sensor. The aSAFE 2.0 approach can alternatively be carried out continuously, with a preset total time, or with a preset number of cycles. However, these modes increase the risk of completely emptying the dropping funnel, which would lead to ventilation of the system and could cause damage to the high vacuum source.

• **Alternative temperature settings**

The temperature settings for the water bath and the circulation thermostat may be lower than 40 °C; however, this may decrease volatile yields and, depending on the type of sample, may lead to a blockage of the valve by solidified fat. Higher temperatures are not recommended as they increase the risk of artifact formation.

• **Higher boiling samples**

The aSAFE 2.0 equipment can, in principle, also handle extracts obtained with higher boiling solvents; however, solvents such as diethyl ether and dichloromethane are usually preferred as their low boiling points facilitate subsequent concentration of the volatile isolate with low thermal impact. Moreover, aqueous-liquid samples can also be directly applied to the aSAFE 2.0 approach, and afterwards, if desired, the aqueous volatile isolate can be extracted with an organic solvent.

• **Time settings**

The software allows a wide range of valve open and valve closed time settings; however, an open time of 0.2 s, corresponding to ∼0.8 mL per portion, is recommended as a standard setting. Higher valve open times may lead to too high portion sizes and increase the risk of a transfer of nonvolatiles to the volatile isolate. On the other hand, valve closed times higher than 20 s can increase yields, but at the cost of a longer overall time required for the aSAFE 2.0 procedure. For low-fat samples, valve closed times below 20 s may be applicable [15]; however, the valve closed time should be long enough to allow complete evaporation of each extract portion as well as complete recondensation/solidification before the next portion enters the evaporation flask.

• **Nonfood materials**

Although the SAFE approach’s main field of application has traditionally been food and beverages, it is not limited to these and can also successfully be used to isolate volatiles from nonfood materials (examples in [[22], [23], [24]]).

## Method validation

Since only a very minor modification was made to the aSAFE glassware to allow the positioning of the endpoint detection sensor between the dropping funnel and the valve, while no modifications were made to the glassware parts under vacuum, we consider the results of the method evaluation reported in the previous aSAFE paper to be fully transferable to the aSAFE 2.0 approach. Detailed information is available in [15].

### Limitations

• **Inhomogeneous extracts**

It is essential that samples applied to the aSAFE 2.0 approach are free of solids. Solid particles may prevent the tight closure of the pneumatic valve, resulting in a continuous influx of sample to the evaporation flask, which can lead to splashing and transfer of nonvolatiles to the volatile isolate as well as to a breakdown of the vacuum. Proper filtration before aSAFE 2.0 is thus essential. Emulsions should be broken, e.g., by centrifugation, followed by careful phase separation.

• **Residual extract volume**

When the endpoint detection sensor senses air below its casing and thus stops the aSAFE 2.0 procedure, a small amount of extract (≤ 0.5 mL) remains trapped upstream of the valve. If exhaustive SAFE processing of the extract is desired, add a few mL of solvent to the dropping funnel and tap the play icon **▹** on the screen of the aSAFE controller to rinse the device.

• **Very highly volatile compounds**

Volatiles with very low boiling points may be poorly recovered. However, compounds with boiling points below the boiling point of the extraction solvent are lost anyway during the subsequent concentration of the volatile isolate, which normally follows SAFE as part of the further workup. These compounds are better covered by complementary headspace sampling techniques [8].

• **High-boiling volatiles**

For compounds with boiling points beyond a certain value, the yields drop below 100%. The boiling point limit depends on the lipid content of the extract. In previous experiments [15], the limits were 270 °C (non-fat extract), 250 °C (low-fat extract with 1 g oil/100 mL), and 180 °C (high-fat extract with 10 g oil/100 mL). However, even then, the yields were still substantially higher than those achieved with the manual SAFE.

• **High-fat samples**

Solvent extracts with a total nonvolatile lipid content exceeding 10% include a high risk of substantial nonvolatile transfer to the volatile isolate. The evaporation cold may also cause solidification of the lipids between the dropping funnel and the thermostated glass parts, leading to a blockage. In this case, the lowest possible valve open time (0.1 s) should be selected; it may also be considered to dilute the extract with solvent and, if necessary, divide the extract before application to the aSAFE 2.0 approach.

## Ethics statements

This work did not include any studies with human or animal subjects, nor data collected from social media platforms.

## Declaration of competing interest

The authors declare the following financial interests/personal relationships which may be considered as potential competing interests: Both authors are shareholders of RS AromaCom, the supplier of the aSAFE 2.0 Kit.

## Data Availability

Data will be made available on request.
